# Comparison of Child Undernutrition Anthropometric Indicators Across 56 Low- and Middle-Income Countries

**DOI:** 10.1001/jamanetworkopen.2022.1223

**Published:** 2022-03-11

**Authors:** Jewel Gausman, Rockli Kim, Zhihui Li, Lucia Tu, Sunil Rajpal, William Joe, S. V. Subramanian

**Affiliations:** 1 Department of Global Health and Population, Harvard T.H. Chan School of Public Health, Boston, Massachusetts; 2 Division of Health Policy and Management, College of Health Science, Korea University, Seoul, South Korea; 3 Interdisciplinary Program in Precision Public Health, Department of Public Health Sciences, Graduate School of Korea University, Seoul, South Korea; 4 Harvard Center for Population and Development Studies, Cambridge, Massachusetts; 5 Department of Social and Behavioral Sciences, Harvard T.H. Chan School of Public Health, Boston, Massachusetts; 6 Vanke School of Public Health, Tsinghua University, Beijing, China; 7 Nathan S. Kline Institute for Psychiatric Research, Orangeburg, New York; 8 Department of Economics, FLAME University, New Delhi, India; 9 Population Research Centre, Institute of Economic Growth, Delhi University Enclave, North Campus, Delhi, India

## Abstract

**Importance:**

The United Nations’ Sustainable Development Goal Target 2.2 seeks to end all forms of malnutrition by 2030 by meeting targets, including the elimination of stunting and wasting in all children younger than 5 years. Such indicators are used to monitor childhood undernutrition but may not provide a complete picture at a population level.

**Objective:**

To compare global estimates of the prevalence of undernutrition using conventional indicators of anthropometric failure (AF; stunting, underweight, and wasting); the Composite Index of Anthropometric Failure (CIAF); and a proposed classification system called Categories of Anthropometric Failure (CAF) as well as to investigate the association of the conventional indicators, CIAF, and CAF with diarrheal disease as an assessment of the validity of each measure.

**Design, Setting, and Participants:**

Cross-sectional study of the prevalence of undernutrition among children in 56 low- and middle-income countries using data from the nationally representative Demographic and Health Surveys. The study included 530 906 children younger than 5 years. Data were collected from June 2005 to December 2018 and analyzed from September 27, 2020, to February 4, 2021.

**Main Outcomes and Measures:**

Undernutrition identified according to conventional indicators (stunting, underweight, and wasting), the CIAF, and the proposed CAF classification system was estimated and compared. Six logistic regression models were used to examine the association between different classifications of anthropometric failure (AF) and morbidity.

**Results:**

A total of 530 906 children (mean [SD] age, 29.0 [17.2] months; 272 355 [51.3%] boys and 258 551 [48.7%] girls) from 56 low- and middle-income countries were included in the analysis. Estimates of undernutrition generated using the conventional indicators of stunting, underweight, and wasting were lower than estimates generated using the CIAF in all countries. The CAF classification system pointed to considerable variation across countries in children with multiple AFs, which does not correspond to the overall prevalence of undernutrition. For example, 7.5% of children in Niger and 7.1% of children in Timor-Leste were stunted, underweight, and wasted, while 56.0% of children in Niger and 71.1% of children in Timor-Leste were undernourished according to the CIAF. In addition, children who had stunting, underweight, and wasting had 1.52 (95% CI, 1.45-1.61) times the odds of diarrhea compared with children who exhibited no AFs.

**Conclusions and Relevance:**

The results of this study highlight the importance of using different approaches to aid understanding of the entire spectrum of AF with regard to research and development of policies and programs to address AF. The use of the CIAF and the CAF classification system may be useful for treatment to prevent AFs and could accelerate progress in meeting targets for the Sustainable Development Goal.

## Key Points

**Question:**

How do different assessments of anthropometric failure in children compare?

**Findings:**

In this cross-sectional study of 530 906 children in 56 low- and middle-income countries, there were substantial differences in the estimates of children experiencing anthropometric failure when using different approaches to measurement. Furthermore, children with simultaneous stunting, underweight, and wasting had significantly higher odds of diarrheal disease compared with children who exhibited no failure.

**Meaning:**

A clearer picture of the prevalence of single and co-occurring anthropometric failure obtained through different methods of measuring undernutrition may accelerate progress toward meeting the targets of the United Nations’ Sustainable Development Goal 2 focused on ending hunger.

## Teaser

This cross-sectional study of children in 56 low- and middle-income countries compares global estimates of undernutrition using conventionally defined classifications of anthropometric failure and alternative classification systems, including the Composite Index of Anthropometric Failure and Categories of Anthropometric Failure.

## Introduction

The United Nations’ Sustainable Development Goal (SDG) 2 aims to eliminate hunger by the year 2030.^1^ Progress toward meeting this goal is defined through a set of targets related to nutritional well-being, agricultural productivity, and sustainability of food systems.^2^ Target 2.2 seeks to end all forms of malnutrition by 2030 by meeting targets including the elimination of stunting and wasting in all children younger than 5 years.^3^ Underweight was used as an indicator to track progress toward ending hunger under the Millennium Development Goals; however, use of this indicator came under scrutiny because it can overstate progress given the increasing dual burden of undernutrition and overnutrition in many countries around the world.^4,5^ Stunting, wasting, and underweight are often labeled collectively as states of anthropometric failure (AF)^6,7^ and serve as proxies for severe malnutrition in the absence of nutritional intake data. Stunting, wasting, and underweight are defined as having a height-for-age *z* score, weight-for-height *z* score, and weight for age *z* score, respectively, of less than −2 SDs from the World Health Organization growth standards reference median.^8^

Although stunting and wasting indeed represent different biological processes, understanding them as separate indicators of nutritional status is a challenge given that they share many of the same causes.^9,10,11^ Anthropometric failure is not static; children pass through different states of AF and may experience 1 or multiple failures at different times in their lives.^10^ An extensive literature review failed to find any independent causes of wasting that were not also associated with stunting.^4,12^ In the last decade, a cyclical association between wasting and stunting has been found. Children with wasting are more likely to develop stunting, and in some places, these conditions may follow seasonal trends and environmental stressors.^10,13,14,15,16^ Generally, stunting is considered to be relatively insensitive to marginal or short-term nutritional insufficiency. In contrast, underweight and wasting may be the result of acute starvation and/or disease, but neither indicator is able to clearly differentiate between recent and chronic nutritional deficiencies.^9,17^ Understanding how the different states of AF are associated with undernutrition is further complicated by the potential role of infectious diseases in reducing appetite, increasing metabolic requirements, and increasing nutrient loss.^18^

Global interest in the co-occurrence of different types of AF is relatively new; the first global studies of which we are aware were published in 2017.^10^ Approaches to measuring childhood nutritional status largely still focus on stunting, underweight, and wasting as individual conditions. Existing World Health Organization indicators for malnutrition do not consider children who meet the criteria for more than 1 category of AF and as such, do not provide a method by which an overall estimate of child malnutrition at a population level can be ascertained.^19^ To provide a more complete picture of undernutrition at the population level, the Comprehensive Index of Anthropometric Failure (CIAF) was proposed as a way to calculate an aggregated estimate of the burden of childhood malnutrition by combining all children experiencing any single type or combination of AF into 1 summary measure^19,20,21,22^; however, as a combined estimate, the CIAF loses the ability to distinguish between children facing different combinations of AF, which may be of specific interest for interventions to reduce the burden of malnutrition.

Previous research suggests that children who experience multiple AFs concurrently, especially those who had stunting, underweight, and wasting simultaneously, may have an elevated risk of morbidity and mortality,^10,19,23,24^ although the cause of co-occurring failures remains poorly understood.^10,23,24^ To address these challenges, we propose the Categories of Anthropometric Failure (CAF) classification system as a way to examine the burden of malnutrition at a population level, which corresponds to the disaggregated categories of AF as described in other studies.^19,24^ The CAF classification system disaggregates the CIAF into all possible combinations of AF: stunting only; underweight only; wasting only; stunting and underweight; wasting and underweight; and stunting, underweight, and wasting. Of note, the combination of stunting and wasting (but not underweight) is theoretically impossible.^23^ The eFigure in the Supplement details the differences between the conventional measures of AF, the CIAF, and the CAF classification system.

The aim of this study is to compare the prevalence of undernutrition using different approaches to measuring AF at the population level. Herein, we present a pooled and cross-country comparison of the prevalence of undernutrition identified by the conventional indicators (stunting, wasting, and underweight), the CIAF, and the CAF classification system using representative data from 56 low- and middle-income countries globally. We also assess the association of the conventional indicators, CIAF, and CAF with diarrheal disease as an assessment of the validity of each measure.

## Methods

### Data Source

In this cross-sectional study, we used data from the most recent Demographic and Health Survey (DHS) in the 56 countries, with surveys conducted since 2010. Demographic and Health Surveys are nationally representative, cross-sectional surveys that have been conducted at regular intervals in more than 85 countries since 1984.^25,26^ The DHS uses a multistage stratified cluster design.^27^ Within each selected household, all children younger than 6 years are eligible for biomarker collection if they are a usual resident or had slept in the household the previous night.^28^ For consistency with international definitions, we include only children 5 years and younger in our analyses. Sampling weights are provided for each survey to calculate nationally representative statistics.

Children’s anthropometric measurements were taken using standardized procedures that are consistent across surveys. Weight was measured using a digital scale, and length was collected by 2 trained individuals using a portable measuring board.^28^ Data were collected from June 2005 to December 2018. The DHS data collection procedures were approved by the ORC Macro International, Inc, Institutional Review Board as well as by the relevant body in each country responsible for approving research studies with human participants. This study was exempt from further review, because it was based on an anonymous public use data set with no identifiable information on the survey participants. Participant oral informed consent was originally obtained at the time of data collection. This report follows the Strengthening the Reporting of Observational Studies in Epidemiology (STROBE) reporting guideline for cross-sectional studies.

### Study Population and Sample

The study population for the pooled analysis included 750 652 children 5 years and younger from 56 countries. We excluded 219 746 children missing anthropometric data. The final sample included 530 906 children.

### Outcomes of Interest

Stunting, wasting, and underweight are the primary outcomes of interest for comparing the prevalence of undernutrition. We defined stunting, wasting, and underweight as having a height-for-age *z* score, weight-for-height *z* score, or weight-for-age *z* score of less than −2 SDs from the World Health Organization’s growth standards reference median.^8^ We calculated the CIAF as the summed total of children who experience any type or combination of AF (specifically those with stunting, underweight, or wasting). For the CAF, we calculated the 6 possible categories of AF separately (stunting only, underweight only, wasting only, stunting and underweight, wasting and underweight, and stunting, underweight, and wasting). In addition, we coded the presence of child diarrheal disease as a binary variable based on whether the child had experienced diarrhea in the last 2 weeks.

### Statistical Analysis

To compare the prevalence of undernutrition using different indicators, we calculated the prevalence of stunting, underweight, and wasting for each country according to the conventional indicators and the percentage of children who had stunting, underweight, or wasting who also exhibited another AF. We then calculated the CIAF for each country and compared it with the percentage of children who had stunting, underweight, and wasting according to the conventional definition. In addition, we calculated the CAF score for each country.

To assess the association of the different indicators of AF considered in this study and diarrheal disease, we conducted a pooled logistic regression analysis, with child diarrheal disease as the outcome measure. We ran 6 different models examining different indicators of AF as the exposure variable; 3 use the conventional measures of AF (stunting, underweight, and wasting), 1 model uses the CIAF, and 1 model uses the CAF on the pooled sample from the 56 countries included in the study. We adjusted for age-, sex-, and country-level fixed effects. *P* < .05 was chosen a priori to represent statistical significance, and 2-sided hypothesis tests were used. Stata, version 14.2 (StataCorp) was used to perform all analyses, and figures were produced using the R statistical program’s package ggplot2 (R Foundation for Statistical Computing).^29,30^ Data were analyzed from September 27, 2020, to February 4, 2021.

## Results

### Pooled, Cross-country Comparison of Undernutrition Prevalence Estimates

**Figure 1.**
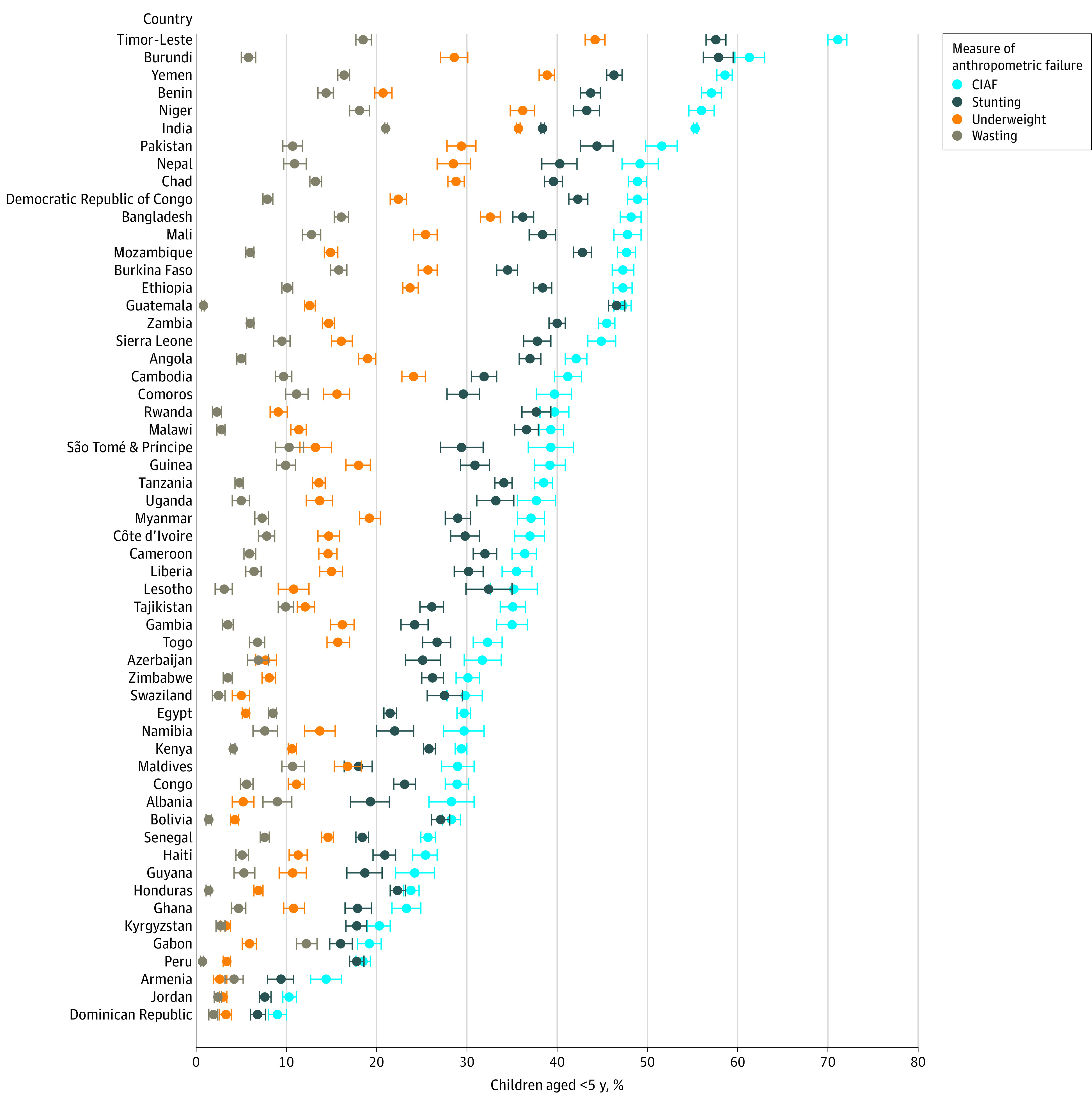
Comparison of Stunting, Underweight, and Wasting with Composite Index of Anthropometric Failure (CIAF) Across 56 Countries Error bars indicate 95% CIs.

Figure 1 shows the prevalence estimates of stunting, underweight, and wasting according to the conventional definitions and the CIAF calculated for the 56 countries included in the study (full results are available in eTable 1 in the Supplement). Burundi had the highest prevalence of stunting (57.9%). Timor-Leste had the highest prevalence of underweight (44.2%). India had the highest prevalence of wasting (21.0%). Timor-Leste had the highest burden of undernutrition as measured by the CIAF (71.1%). Although the overall prevalence of stunting in Timor-Leste and Burundi was similar (57.6% and 57.9%, respectively), the percentages of children who were underweight and wasting were significantly lower in Burundi than in Timor-Leste (12.7% lower and 15.7% lower, respectively). When comparing the aggregate burden of undernutrition in each country as measured by the CIAF, the CIAF suggested a 10% higher prevalence of undernutrition in Timor-Leste than in Burundi, a difference that is not directly ascertainable from looking at each conventional indicator alone. In the case of India, the substantial overlap in children experiencing multiple AFs is evident when comparing the prevalence of stunting (38.4%), underweight (35.7%), and wasting (21.0%) with the aggregate estimate provided by the CIAF (55.2%).

**Figure 2.**
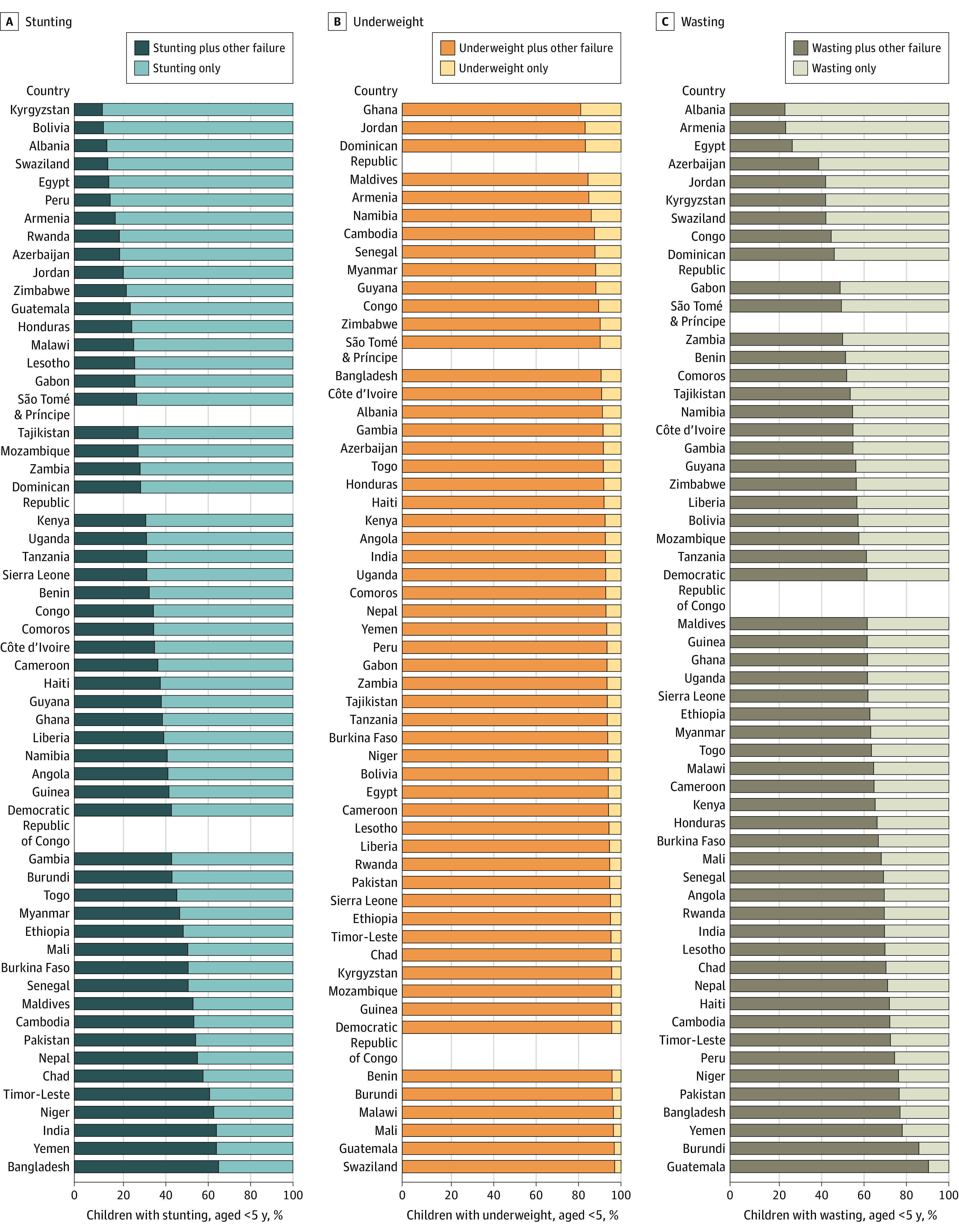
Percentage of Children With Stunting, Underweight, and Wasting Who Had Only 1 Anthropometric Failure or Experienced 1 Anthropometric Failure in Combination With Another Failure in 56 Countries

Figure 2 shows the percentage of children with stunting, underweight, or wasting with 1 or concurrent AFs (full estimates are available in eTable 2 in the Supplement) to illustrate the wide variation in the co-occurrence of AF in children globally. There were 13 countries in which more than 50% of children with stunting had at least 1 additional concurrent AF, and there were 7 countries in which less than 20% of children with stunting had an additional AF. Most children with underweight also experience another type of failure, but there still remains an important percentage of children with underweight with no other concurrent AF. Ghana had the highest percentage of children who were only underweight without an additional, concurrent AF (18.4%), and Guatemala had the lowest (3.1%). Guatemala had the highest percentage of children who experienced wasting in combination with another type of AF (90.7%), whereas only 25.4% of children who experienced wasting in Armenia had another concurrent failure.

**Figure 3.**
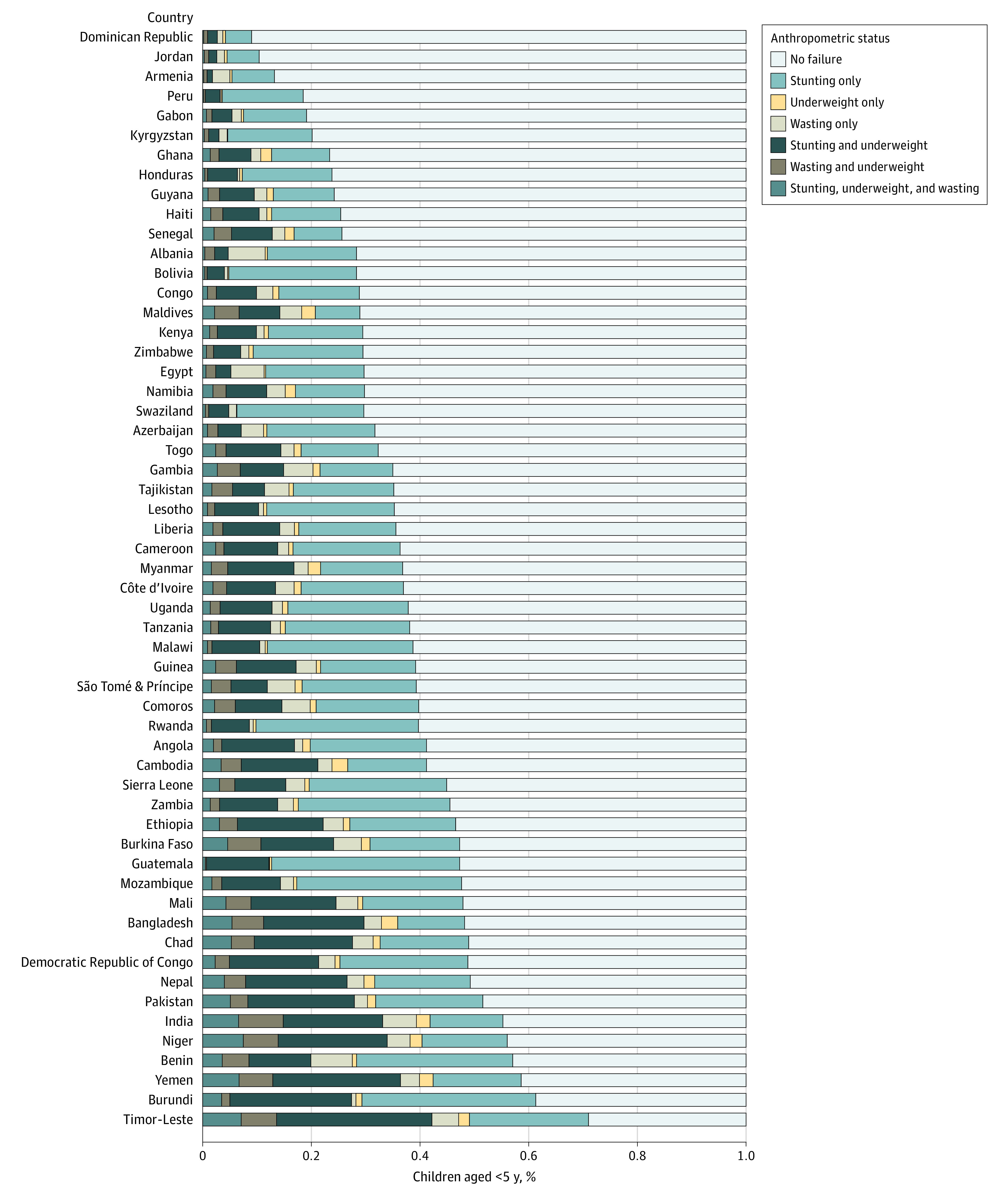
Categories of Anthropometric Failure Across 56 Countries

Figure 3 presents the prevalence of each disaggregated type of AF as defined by the CAF classification system across all 56 countries included in the study (full estimates are available in eTable 3 in the Supplement). Among the 29 countries where the prevalence of stunting was greater than 30%, the percentage of children who had stunting with other concurrent forms of AF varies considerably. Within these countries, the percentage of children who were stunting and underweight ranged from 7.0% in Rwanda to 28.6% in Timor-Leste, and the percentage of children who had stunting, underweight, and wasting ranged from 0.5% in Guatemala to 7.5% in Niger. However, in other countries, the prevalence of stunting was lower, but the proportion of children who experienced stunting in combination with another AF was substantial. For example, in the Republic of Maldives and Senegal, the prevalence of stunting was 18.0% and 18.4% respectively, but approximately 40% of those children were stunting and underweight. In Gambia, the prevalence of stunting was 24.2%, but 11.1% of those children with stunting, underweight, and wasting. Among the countries where more than 10% of children were wasting, the proportion of children who had stunting, underweight, and wasting ranged from 1.6% in São Tomé and Príncipe to 7.5% in Niger. Again, other countries with a lower prevalence of wasting also had a substantial proportion of children who had stunting, underweight, and wasting. For instance, 5.8% of children in Burundi experienced wasting, but 63.7% of those children had stunting, underweight, and wasting.

### Association of Conventional Indicators of AF, CIAF, and CAF With Diarrheal Disease

The Table presents the results of 6 logistic regression models showing the association of the 3 conventional indicators of AF, the CIAF, and the CAF with child diarrheal disease. All indicators of AF were associated with increased odds of diarrhea. Of the conventional indicators of AF, model 3 (child is underweight vs not underweight) showed that children who were underweight compared with those who were not had the highest odds (1.32; 95% CI, 1.28-1.36) of having experienced diarrhea in the last 2 weeks compared with the estimates obtained from the models including the other forms of AF (model 2 [stunting vs no stunting] and model 4 [wasting vs no wasting]). Model 6 (CAF) included the different combinations of AF as measured by the CAF. In this model, children who had stunting, underweight, and wasting had 1.52 (95% CI, 1.45-1.61) times the odds of diarrhea compared with children who exhibited no AFs, which was the highest among the different combinations of failure included in the CAF.

**Table.** Pooled, Logistic Regression of Child Diarrheal Disease on Different Categorizations of Anthropometric Failure Across 56 Countries Adjusted for Age, Sex, and Country-Level Fixed Effects (N = 530 906)

Variable	OR (95% CI)^a^					
	Model 1	Model 2	Model 3	Model 4	Model 5	Model 6
No failure (reference: any failure)	NA	NA	NA	NA	NA	NA
Stunting (reference: not Stunting)	1.23 (1.20-1.26)	NA	NA	1.14 (1.11-1.17)	NA	NA
Underweight (reference: not underweight)	NA	1.32 (1.28-1.36)	NA	1.22 (1.18-1.27)	NA	NA
Wasting (reference: no wasting)	NA	NA	1.13 (1.09-1.16)	1.0 (0.99-1.07)	NA	NA
CIAF (reference: no failure)	NA	NA	NA	NA	1.22 (1.19-1.25)	NA
Stunting only (reference: no failure)	NA	NA	NA	NA	NA	1.14 (1.10-1.17)
Underweight only	NA	NA	NA	NA	NA	1.21 (1.11-1.32)
Wasting only	NA	NA	NA	NA	NA	0.94 (0.89-1.00)
Stunting and underweight	NA	NA	NA	NA	NA	1.36 (1.31-1.40)
Underweight and wasting	NA	NA	NA	NA	NA	1.28 (1.21-1.34)
Stunting, underweight and wasting	NA	NA	NA	NA	NA	1.52 (1.45-1.61)

Abbreviations: CIAF, Composite Index of Anthropometric Failure; NA, not applicable; OR, odds ratio.

^a^ Model 1: any failure vs no failure. Model 2: stunting vs no stunting. Model 3: underweight vs not underweight. Model 4: wasting vs no wasting. Model 5: CIAF. Model 6: Categories of Anthropometric Failure.

## Discussion

The results of this study provide important considerations for how undernutrition, as defined by AF, is measured globally, which may have implications for achieving SDG Target 2.2. As of 2015, only 16.5% out of 188 countries had eliminated stunting, the same percentage that had eliminated wasting.^31^ First, the results suggest that the picture of AF across countries is largely dependent on the measurement approach used. Second, there is substantial overlap between the 3 conventional indicators used to assess undernutrition at a population level and estimates of undernutrition using these indicators vary considerably across countries, thus potentially blurring international comparisons of child nutritional status. Furthermore, focusing only on the overall prevalence of stunting, underweight, and wasting without disaggregation may obscure the proportion of children in a population most at risk for poor health outcomes owing to multiple, simultaneous AFs.^19,32^ Third, results of the present study suggest that further examination of the association between multiple, concurrent AFs, as defined by the CAF, and other forms of child morbidity may be beneficial to programs seeking to address undernutrition, but more research is needed to further elucidate this association. Without examining the co-occurrence of different types of AF, policy and intervention may be somewhat misaligned with the burden of disease.

The use of distinct indicators for program monitoring may also give an incomplete picture of progress. Changes observed over time in stunting, underweight, or wasting can be difficult to interpret because they may give mixed messages on both the rate and direction of change in undernutrition.^33^ For instance, Nandy et al^33^ highlighted data from 2 consecutive Demographic and Health Surveys in Zimbabwe, which provided conflicting information about changes in child nutritional status: the number of children who exhibited stunting and wasting increased between surveys, but the number of underweight children declined. Part of the difficulty in interpreting these indicators may be owing to the considerable overlap in the population experiencing multiple, concurrent failures.^20^

**Figure 4.**
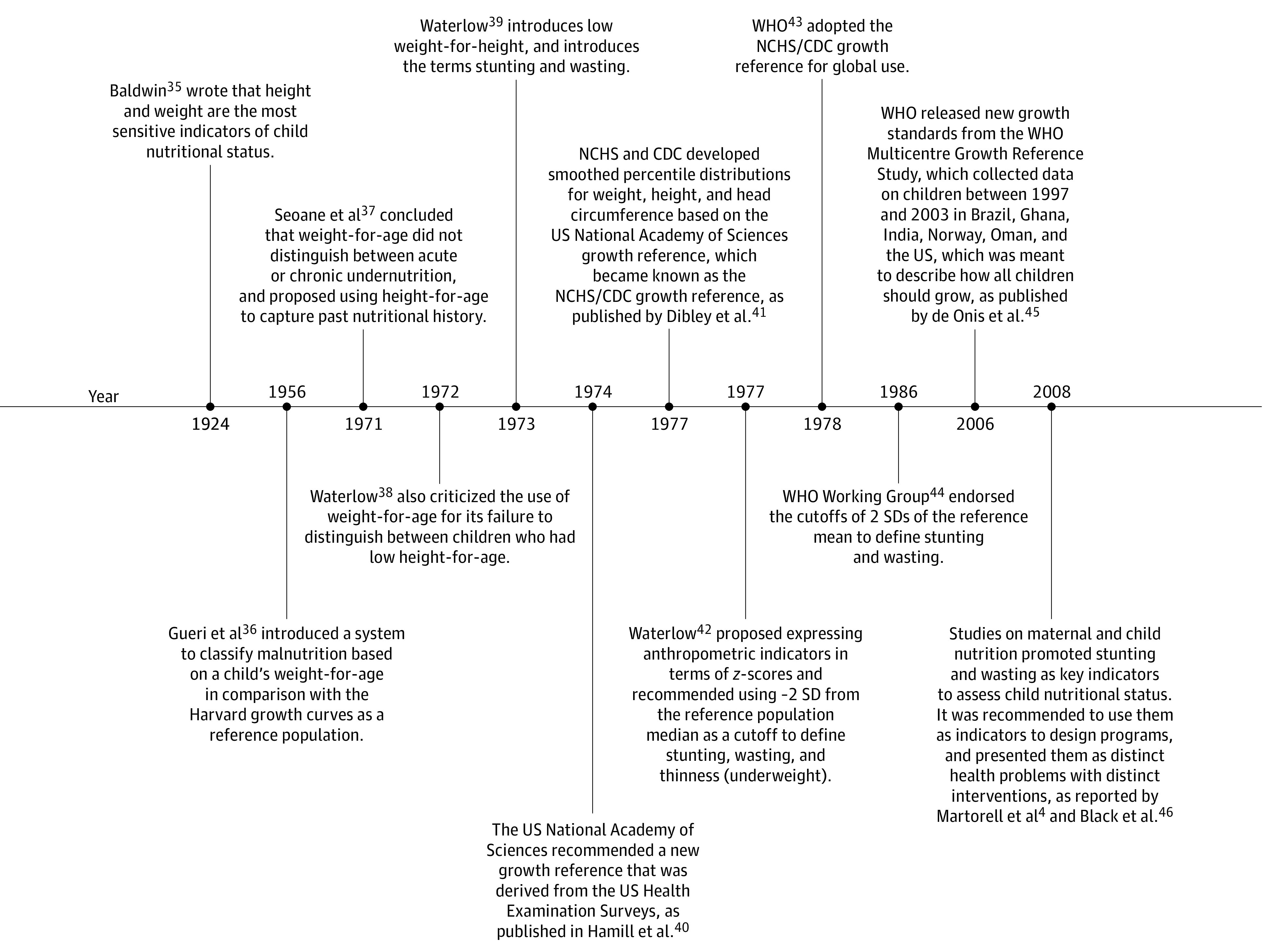
Historical Evolution of Measures of Anthropometric Failure Since 1900 CDC indicates US Centers for Disease Control and Prevention; NCHS, National Center for Health Statistics; WHO, World Health Organization.

The current focus on the 3 conventional indicators of undernutrition represents decades of work to build evidence and consensus around these measures. The use of anthropometry to assess malnutrition grew from early efforts to create an internationally comparable classification system for estimating the burden of undernutrition at a population level.^34^ A timeline showing the evolution of studies^35,36,37,38,39,40,41,42,43,44,45,46^ using anthropometry in assessing undernutrition is shown in Figure 4. Low height-for-age and weight-for-height were proposed as measures of undernutrition in the early 1970s, which were subsequently termed stunting and wasting.^35,36^ The use of −2 SD from the reference population median as a cutoff was introduced in 1977 and endorsed by the World Health Organization in 1986,^37,44^ although it was noted at the time that the choice of −2 SD as the cutoff was somewhat arbitrary.^37,47^ The use of a statistically determined cutoff has since been questioned given that there is no biological basis for its definition as a clear threshold, and the association between anthropometry and poor health operates along a continuous gradient.^48^ In 2008, studies on maternal and child undernutrition promoted the use of stunting and wasting to assess child nutritional status globally and presented them as distinct concerns with distinct interventions.^4,36^

The use of the CIAF provides a simplified way to assess undernutrition by offering a single, comprehensive estimate; however, it does not provide information on children experiencing multiple AFs. Therefore, the CAF may be a useful tool for policy makers and interventionists to better prevent and address specific combinations of AF, especially if coupled with a deeper understanding of the differing causes of different types of concurrent failure. It should be noted that both the CIAF and the CAF rely on different configurations of the conventional, widely used indicators of AF. A detailed comparison of the advantages and disadvantages of each approach is provided in eTable 4 in the Supplement. Future research should question whether all categories of AF are truly useful in assessing undernutrition within a population and their association with morbidity and mortality.^19^

The results of this study highlight the importance of considering different methods of measuring AF to aid understanding of the entire spectrum of AF. By examining the composition of the different categories of AF within a population, new insights can be gained about the burden of disease, which may lead to a prioritization of different programmatic and policy approaches in countries with similar overall burdens of AF. For example, the overall prevalence of AF as measured by stunting and the CIAF is similar in Mali and Mozambique; however, the CAF provides important insight into the composition of AF within each country, suggesting a much larger burden of multiple AFs in children in Mali than in Mozambique. Thus, despite a similar overall burden of AF as defined through conventional indicators, the CAF illustrates that different programmatic approaches may be necessary in each country to most effectively reduce AF given the dramatically different burden of disease when considering concurrency.

The use of the CIAF and the CAF may be useful in responding to calls to shift focus from treatment to prevention of undernutrition^10^ by contributing to the discourse on whether to prioritize individual vs community intervention. Previous research on disaggregated measures of AF derived from the CIAF in India found that children who had stunting, underweight, and wasting tended to live in the poorest households.^19^ Because economically disadvantaged children are more likely to have multiple AFs simultaneously, the presence of multiple vs singular AFs may better distinguish between chronic and acute undernutrition than the traditional distinction between stunting vs wasting. More research using the CAF could help elucidate individual and ecological factors of multiple failures to better design interventions to prevent AFs altogether or to prevent children with 1 failure from developing multiple failures.

Progress toward reducing morbidity and mortality among children who have AF may be limited if interventions are successful at reducing stunting or wasting individually without addressing the complex interplay between the co-occurrence of the different categories of AF.^10^ Furthermore, given the cyclic association between stunting and wasting, addressing them as separate conditions may limit the effectiveness of programmatic efforts that view them as distinct; however, different states of AF respond to treatment differently.^10^ In particular, there are many causes of stunting, and the results of the present study emphasize that not all countries in which a large proportion of children with stunting also have large proportions of children with wasting. Thus, focusing prevention efforts to specifically address children with the highest risk of morbidity and mortality associated with AF may allow country programs to better focus efforts on reducing adverse health outcomes among children at highest risk. In particular, interventions designed to address wasting in populations where wasting is an important factor of stunting may focus intervention efforts on specific seasons or addressing risks specific to children by sex, socioeconomic status, or maternal characteristics.^16^ Thus, prevention efforts that consider the complex and upstream common causes of concurrent failures may ultimately have a more substantial impact on preventing children from experiencing multiple forms of AF as well as the poor health outcomes associated with concurrent failures than those that focus on singular etiologic pathways.

### Limitations

This study has several important limitations. First, the data presented are cross-sectional, and thus, we cannot account for children who move between categories of AF over time. Second, the results may be subject to measurement error that could affect the comparability of results between countries. Furthermore, approximately 29% of the children in the initial sample were missing anthropometric data across all countries. Because we did not find any patterns related to missing data, we believe that the data are missing at random; thus, we do not believe that the missing data bias our results. Third, we focused the analysis on undernutrition and did not consider obesity; overweight was added to the CIAF in 2007.^21^

## Conclusions

In this study, the CAF found considerable variation across countries in children with multiple AFs that did not correspond to the overall prevalence of undernutrition. Current progress is limited in eliminating stunting and wasting. Progress must improve if countries around the globe are to meet the targets set forth in the SDG 2. Examining different methods of measuring undernutrition may contribute to a deeper understanding of the spectrum of AF as well as inform policies and programs to address AFs through prevention and treatment. In turn, accelerating progress toward meeting the SDG 2.2 target may be possible.

## Notes

Supplement. **eTable 1.** Percentage Stunted, Underweight, Wasted and the Composite Index of Anthropometric Failure Among Children Under 5 Years Across 56 Countries **eTable 2.** Percentage of Children Under 5 Years With Single and Multiple Failure Among Those Experiencing Stunting, Underweight and Wasting Across 56 Countries **eTable 3.** Percentage of Children Under 5 Experiencing Individual and Concurrent Anthropometric Failures According to the Categories of Anthropometric Failure Across 56 Countries **eTable 4.** Summary of Current Approaches for Estimating Undernutrition and Their Advantages and Disadvantages **eFigure.** Comparison of Different Approaches to Measuring Undernutrition by Anthropometric Failure (AF): Conventional Categorization of AF, the Composite Index of AF (CIAF) and the Categories of AF (CAF)

## References

[1] United Nations. Sustainable Development Goal 2. Published 2019. Accessed January 30, 2020. https://sdgs.un.org/goals/goal2

[2] Sustainable Development Goals Knowledge Platform. Sustainable Development Goal 2. United Nations. Published 2019. Accessed November 4, 2019. https://sustainabledevelopment.un.org/sdg2

[3] Lee BX, Kjaerulf F, Turner S, . Transforming our world: implementing the 2030 agenda through sustainable development goal indicators. J Public Health Policy. 2016;37(1)(suppl 1):13-31. doi:10.1057/s41271-016-0002-7 27638240

[4] Martorell R, Young MF. Patterns of stunting and wasting: potential explanatory factors. Adv Nutr. 2012;3(2):227-233. doi:10.3945/an.111.001107 22516733 PMC3648726

[5] Uauy R, Kain J, Mericq V, Rojas J, Corvalán C. Nutrition, child growth, and chronic disease prevention. Ann Med. 2008;40(1):11-20. doi:10.1080/07853890701704683 18246473

[6] Mejía-Guevara I, Corsi DJ, Perkins JM, Kim R, Subramanian SV. Variation in anthropometric status and growth failure in low-and middle-income countries. Pediatrics. 2018;141(3):e20172183. doi:10.1542/peds.2017-2183 29472493

[7] Subramanian SV, Ackerson LK, Davey Smith G, John NA. Association of maternal height with child mortality, anthropometric failure, and anemia in India. JAMA. 2009;301(16):1691-1701. doi:10.1001/jama.2009.548 19383960 PMC3095774

[8] World Health Organization. WHO Child Growth Standards: Length/Height for age, Weight-For-Age, Weight-For-Length, Weight-For-Height and Body Mass Index-for-Age, Methods and Development. World Health Organization; 2006.

[9] World Health Organization. Physical status: the use of and interpretation of anthropometry, report of a WHO Expert Committee. Accessed February 2, 2022. https://apps.who.int/iris/bitstream/handle/10665/37003/W?sequence=1 8594834

[10] Wells JCK, Briend A, Boyd EM, . Beyond wasted and stunted-a major shift to fight child undernutrition. Lancet Child Adolesc Health. 2019;3(11):831-834. doi:10.1016/S2352-4642(19)30244-5 31521500

[11] Collins S, Dent N, Binns P, Bahwere P, Sadler K, Hallam A. Management of severe acute malnutrition in children. Lancet. 2006;368(9551):1992-2000. doi:10.1016/S0140-6736(06)69443-9 17141707

[12] Briend A, Khara T, Dolan C. Wasting and stunting: similarities and differences: policy and programmatic implications. Food Nutr Bull. 2015;36(1)(suppl):S15-S23. doi:10.1177/15648265150361S103 25902610

[13] Maleta K, Virtanen SM, Espo M, Kulmala T, Ashorn P. Seasonality of growth and the relationship between weight and height gain in children under three years of age in rural Malawi. Acta Paediatr. 2003;92(4):491-497. doi:10.1111/j.1651-2227.2003.tb00584.x 12801119

[14] Victora CG, Adair L, Fall C, ; Maternal and Child Undernutrition Study Group. Maternal and child undernutrition: consequences for adult health and human capital. Lancet. 2008;371(9609):340-357. doi:10.1016/S0140-6736(07)61692-4 18206223 PMC2258311

[15] Schoenbuchner SM, Dolan C, Mwangome M, . The relationship between wasting and stunting: a retrospective cohort analysis of longitudinal data in Gambian children from 1976 to 2016. Am J Clin Nutr. 2019;110(2):498-507. doi:10.1093/ajcn/nqy326 30753251 PMC6669055

[16] Thurstans S, Sessions N, Dolan C, . The relationship between wasting and stunting in young children: A systematic review. Matern Child Nutr. 2022;18(1):e13246. doi:10.1111/mcn.13246 34486229 PMC8710094

[17] Corsi DJ, Subramanyam MA, Subramanian SV. Commentary: measuring nutritional status of children. Int J Epidemiol. 2011;40(4):1030-1036. doi:10.1093/ije/dyr108 21724577

[18] Caulfield LE, Richard SA, Rivera JA, Musgrove P, Black RE. Stunting, wasting, and micronutrient deficiency disorders. In: Disease Control Priorities in Developing Countries. 2nd edition. The International Bank for Reconstruction and Development/The World Bank; 2006. 21250337

[19] Nandy S, Irving M, Gordon D, Subramanian SV, Smith GD. Poverty, child undernutrition and morbidity: new evidence from India. Bull World Health Organ. 2005;83(3):210-216. 15798845 PMC2624218

[20] Svedberg P. How many people are malnourished? Annu Rev Nutr. 2011;31:263-283. doi:10.1146/annurev-nutr-081810-160805 21756133

[21] Nandy S, Svedberg P. The Composite Index of Anthropometric Failure (CIAF): An alternative indicator for malnutrition in young children. In: Handbook of Anthropometry. Springer; 2012:127-137.

[22] Svedberg P. Poverty and Undernutrition: Theory, Measurement, and Policy. Oxford University Press; 2000.

[23] Myatt M, Khara T, Schoenbuchner S, . Children who are both wasted and stunted are also underweight and have a high risk of death: a descriptive epidemiology of multiple anthropometric deficits using data from 51 countries. Arch Public Health. 2018;76(1):28. doi:10.1186/s13690-018-0277-1 30026945 PMC6047117

[24] McDonald CM, Olofin I, Flaxman S, ; Nutrition Impact Model Study. The effect of multiple anthropometric deficits on child mortality: meta-analysis of individual data in 10 prospective studies from developing countries. Am J Clin Nutr. 2013;97(4):896-901. doi:10.3945/ajcn.112.047639 23426036

[25] Corsi DJ, Neuman M, Finlay JE, Subramanian SV. Demographic and health surveys: a profile. Int J Epidemiol. 2012;41(6):1602-1613. doi:10.1093/ije/dys184 23148108

[26] Rutstein SO. Guide to DHS Statistics. 2006. Accessed February 2, 2022. https://citeseerx.ist.psu.edu/viewdoc/download?doi=10.1.1.431.8235&rep=rep1&type=pdf

[27] ICF International. Demographic and Health Survey Sampling and Household Listing Manual. Measure DHS; 2012.

[28] ICF International. *MEASURE DHS Biomarker Field Manual*. Calverton, MD: ICF International; 2012.

[29] Stata Statistical Software: Release 14 [computer program]. College Station, TX: StataCorp LP.; 2015.

[30] Wickham H. ggplot2: Elegant Graphics for Data Analysis. Springer; 2016.

[31] Lim SS, Allen K, Bhutta ZA, ; GBD 2015 SDG Collaborators. Measuring the health-related Sustainable Development Goals in 188 countries: a baseline analysis from the Global Burden of Disease Study 2015. Lancet. 2016;388(10053):1813-1850. doi:10.1016/S0140-6736(16)31467-2 27665228 PMC5055583

[32] Khara T, Mwangome M, Ngari M, Dolan C. Children concurrently wasted and stunted: a meta-analysis of prevalence data of children 6-59 months from 84 countries. Matern Child Nutr. 2018;14(2):e12516. doi:10.1111/mcn.12516 28944990 PMC5901398

[33] Nandy S, Miranda JJ. Overlooking undernutrition? using a composite index of anthropometric failure to assess how underweight misses and misleads the assessment of undernutrition in young children. Soc Sci Med. 2008;66(9):1963-1966. doi:10.1016/j.socscimed.2008.01.021 18299166 PMC2685640

[34] Waterlow JC. Classification and definition of protein-energy malnutrition. Monogr Ser World Health Organ. 1976;62(62):530-555. 824854

[35] Baldwin BT. The use and abuse of weight-height-age tables as indexes of health and nutrition. JAMA. 1924;82(1):1-4. doi:10.1001/jama.1924.02650270005001

[36] Gueri M, Gurney JM, Jutsum P. The Gomez classification: time for a change? Bull World Health Organ. 1980;58(5):773-777. 6975186 PMC2395976

[37] Seoane N, Latham MC. Nutritional anthropometry in the identification of malnutrition in childhood. J Trop Pediatr Environ Child Health. 1971;17(3):98-104. doi:10.1093/tropej/17.3.98 5210145

[38] Waterlow JC. Classification and definition of protein-calorie malnutrition. BMJ. 1972;3(5826):566-569. doi:10.1136/bmj.3.5826.566 4627051 PMC1785878

[39] Waterlow JC. Note on the assessment and classification of protein-energy malnutrition in children. Lancet. 1973;2(7820):87-89. doi:10.1016/S0140-6736(73)93276-5 4123633

[40] Hamill PV, Drizd TA, Johnson CL, Reed RB, Roche AF. NCHS Growth Curves for Children Birth-18 Years. Department of Health Education and Welfare; 1977. 611680

[41] Dibley MJ, Goldsby JB, Staehling NW, Trowbridge FL. Development of normalized curves for the international growth reference: historical and technical considerations. Am J Clin Nutr. 1987;46(5):736-748. doi:10.1093/ajcn/46.5.736 3314468

[42] Waterlow JC, Buzina R, Keller W, Lane JM, Nichaman MZ, Tanner JM. The presentation and use of height and weight data for comparing the nutritional status of groups of children under the age of 10 years. Bull World Health Organ. 1977;55(4):489-498. 304391 PMC2366685

[43] Ogden CL, Kuczmarski RJ, Flegal KM, . Centers for Disease Control and Prevention 2000 growth charts for the United States: improvements to the 1977 National Center for Health Statistics version. Pediatrics. 2002;109(1):45-60. doi:10.1542/peds.109.1.45 11773541

[44] WHO Working Group. Use and interpretation of anthropometric indicators of nutritional status. Bull World Health Organ. 1986;64(6):929-941. 3493862 PMC2490974

[45] de Onis M, Onyango AW, Borghi E, Garza C, Yang H; WHO Multicentre Growth Reference Study Group. Comparison of the World Health Organization (WHO) Child Growth Standards and the National Center for Health Statistics/WHO international growth reference: implications for child health programmes. Public Health Nutr. 2006;9(7):942-947. doi:10.1017/PHN20062005 17010261

[46] Black RE, Victora CG, Walker SP, ; Maternal and Child Nutrition Study Group. Maternal and child undernutrition and overweight in low-income and middle-income countries. Lancet. 2013;382(9890):427-451. doi:10.1016/S0140-6736(13)60937-X 23746772

[47] Waterlow JC. Protein-energy malnutrition: the nature and extent of the problem. Clin Nutr. 1997;16(suppl 1):3-9. doi:10.1016/S0261-5614(97)80043-X 16844615

[48] de Onis M. Measuring nutritional status in relation to mortality. Bull World Health Organ. 2000;78(10):1271-1274. 11100621 PMC2560620

