# Supplementary material for: Comparison of Child Undernutrition Anthropometric Indicators Across 56 Low- and Middle-Income Countries
Source: JAMA Netw Open. 2022 Mar 11;5(3):e221223. doi: 10.1001/jamanetworkopen.2022.1223 (PMC8917428; doi:10.1001/jamanetworkopen.2022.1223)
Supplement: Supplement. — eTable 1. Percentage Stunted, Underweight, Wasted and the Composite Index of Anthropometric Failure Among Children Under 5 Years Across 56 Countries eTable 2. Percentage of Children Under 5 Years With Single and Multiple Failure Among Those Experiencing Stunting, Underweight and Wasting Across 56 Countries eTable 3. Percentage of Children Under 5 Experiencing Individual and Concurrent Anthropometric Failures According to the Categories of Anthropometric Failure Across 56 Countries eTable 4. Summary of Current Approaches for Estimating Undernutrition and Their Advantages and Disadvantages eFigure. Comparison of Different Approaches to Measuring Undernutrition by Anthropometric Failure (AF): Conventional Categorization of AF, the Composite Index of AF (CIAF) and the Categories of AF (CAF) [file jamanetwopen-e221223-s001.pdf]

Supplementary Online Content

Gausman J, Kim R, Li Z, et al. Comparison of child undernutrition anthropometric indicators across 56 low- and middle-income countries. *JAMA Netw Open.* 2022;5(3):e221223. doi:10.1001/jamanetworkopen.2022.1223

eTable 1. Percentage Stunted, Underweight, Wasted and the Composite Index of Anthropometric Failure Among Children Under 5 years Across 56 Countries
eTable 2. Percentage of Children Under 5 Years with Single and Multiple Failure Among Those Experiencing Stunting, Underweight and Wasting Across 56 Countries
eTable 3. Percentage of Children Under 5 Experiencing Individual and Concurrent Anthropometric Failures According to the Categories of Anthropometric Failure Across 56 Countries
eTable 4. Summary of Current Approaches for Estimating Undernutrition and Their Advantages and Disadvantages
eFigure. Comparison of Different Approaches to Measuring Undernutrition by Anthropometric Failure (AF): Conventional Categorization of AF, the Composite Index of AF (CIAF) and the Categories of AF (CAF)

This supplementary material has been provided by the authors to give readers additional information about their work.

© 2022 Gausman J et al. *JAMA Network Open.*

eTable 1. Percentage Stunted, Underweight, Wasted and the Composite Index of Anthropometric Failure Among Children Under 5 years Across 56 Countries

Country	Stunting prevalence (%)	Underweight prevalence (%)	Wasting prevalence (%)	CIAF
**Global**	**34.9(34.8, 35.0)**	**24.7(24.6, 24.8)**	**13.3(13.2, 13.4)**	**45.8(45.6, 45.9)**
Albania	19.3(17.1, 21.4)	5.2(4.0, 6.4)	9.0(7.4, 10.6)	28.3(25.8, 30.8)
Angola	37.0(35.8, 38.2)	19.0(18.0, 19.9)	5.0(4.5, 5.5)	42.1(40.9, 43.3)
Armenia	9.4(7.9, 10.8)	2.6(1.9, 3.4)	4.2(3.2, 5.2)	14.4(12.7, 16.1)
Azerbaijan	25.1(23.2, 27.1)	7.7(6.6, 8.9)	6.9(5.7, 8.0)	31.7(29.7, 33.8)
Benin	43.7(42.6, 44.8)	20.7(19.8, 21.7)	16.1(15.3, 16.9)	57.1(56.0, 58.2)
Bangladesh	36.2(35.1, 37.4)	32.6(31.5, 33.7)	14.4(13.5, 15.2)	48.2(47.0, 49.3)
Bolivia	27.1(26.1, 28.1)	4.3(3.8, 4.7)	1.4(1.1, 1.7)	28.3(27.3, 29.3)
Burkina Faso	34.5(33.3, 35.6)	25.7(24.6, 26.7)	15.8(14.9, 16.7)	47.3(46.1, 48.5)
Burundi	57.9(56.2, 59.5)	28.6(27.1, 30.1)	5.8(5.0, 6.6)	61.3(59.7, 63.0)
Cambodia	31.9(30.5, 33.3)	24.1(22.8, 25.4)	9.7(8.8, 10.6)	41.2(39.7, 42.7)
Cameroon	32.0(30.7, 33.3)	14.6(13.6, 15.6)	5.9(5.3, 6.6)	36.4(35.0, 37.7)
Chad	39.6(38.6, 40.6)	28.8(27.9, 29.7)	13.2(12.6, 13.9)	48.9(47.9, 49.9)
Comoros	29.6(27.8, 31.4)	15.6(14.1, 17.0)	11.1(9.9, 12.4)	39.7(37.7, 41.6)
Congo	23.1(21.9, 24.3)	11.1(10.2, 12.0)	5.6(4.9, 6.3)	28.9(27.6, 30.2)
Cote d'Ivoire	29.8(28.2, 31.4)	14.7(13.5, 15.9)	7.8(6.9, 8.7)	37.0(35.3, 38.6)
DRC[§]	42.3(41.3, 43.4)	22.4(21.5, 23.3)	7.9(7.4, 8.5)	48.9(47.8, 50.0)
DR[§§]	6.8(6.0, 7.7)	3.3(2.6, 3.9)	1.9(1.4, 2.4)	9.0(8.0, 10.0)
Egypt	21.5(20.8, 22.2)	5.5(5.1, 5.9)	8.5(8.0, 8.9)	29.7(28.9, 30.4)
Ethiopia	38.4(37.4, 39.4)	23.7(22.9, 24.6)	10.1(9.5, 10.7)	47.3(46.2, 48.3)
Gambia	24.2(22.7, 25.7)	16.2(14.9, 17.5)	12.2(11.1, 13.4)	35.0(33.3, 36.7)
Gabon	16.0(14.8, 17.3)	5.9(5.1, 6.7)	3.5(2.9, 4.1)	19.2(17.9, 20.5)
Ghana	17.9(16.5, 19.4)	10.8(9.7, 12.0)	4.7(3.9, 5.5)	23.3(21.7, 24.9)
Guatemala	46.6(45.7, 47.5)	12.6(12.0, 13.2)	0.8(0.6, 0.9)	47.3(46.3, 48.2)
Guinea	30.9(29.3, 32.5)	18.0(16.6, 19.3)	9.9(8.9, 11.0)	39.2(37.5, 40.9)
Guyana	18.7(16.7, 20.6)	10.7(9.2, 12.2)	5.3(4.2, 6.5)	24.2(22.1, 26.4)
Haiti	20.9(19.6, 22.1)	11.3(10.3, 12.3)	5.1(4.4, 5.8)	25.4(24.0, 26.7)
Honduras	22.3(21.5, 23.2)	6.9(6.4, 7.4)	1.4(1.1, 1.6)	23.8(23.0, 24.7)
India	38.4(38.2, 38.6)	35.7(35.5, 35.9)	21.0(20.9, 21.2)	55.3(55.1, 55.5)
Jordan	7.6(7.0, 8.3)	3.0(2.6, 3.4)	2.4(2.0, 2.8)	10.3(9.6, 11.1)
Kenya	25.8(25.2, 26.5)	10.6(10.2, 11.1)	4.1(3.8, 4.3)	29.4(28.7, 30.0)
Kyrgyzstan	17.8(16.6, 18.9)	3.3(2.7, 3.8)	2.7(2.2, 3.2)	20.3(19.0, 21.5)
Lesotho	32.4(29.9, 35.0)	10.8(9.1, 12.5)	3.1(2.1, 4.0)	35.2(32.6, 37.8)
Liberia	30.2(28.6, 31.8)	15.0(13.7, 16.2)	6.4(5.5, 7.2)	35.5(33.9, 37.2)
Malawi	36.6(35.3, 37.9)	11.4(10.5, 12.2)	2.8(2.3, 3.2)	39.3(38.0, 40.7)

© 2022 Gausman J et al. *JAMA Network Open.*

Maldives	18.0(16.4, 19.5)	16.8(15.3, 18.3)	10.7(9.5, 12.0)	29.0(27.2, 30.8)
Mali	38.4(36.9, 39.8)	25.4(24.1, 26.7)	12.8(11.8, 13.8)	47.8(46.3, 49.3)
Mozambique	42.8(41.8, 43.8)	14.9(14.2, 15.7)	6.0(5.5, 6.4)	47.7(46.7, 48.7)
Myanmar	29.0(27.6, 30.4)	19.2(18.1, 20.4)	7.3(6.5, 8.0)	37.1(35.6, 38.6)
Namibia	22.0(20.0, 24.1)	13.7(12.0, 15.4)	7.6(6.3, 9.0)	29.7(27.4, 31.9)
Nepal	40.3(38.3, 42.2)	28.5(26.7, 30.4)	10.9(9.7, 12.2)	49.2(47.2, 51.2)
Niger	43.3(41.8, 44.7)	36.2(34.8, 37.5)	18.1(17.0, 19.2)	56.0(54.6, 57.4)
Pakistan	44.4(42.6, 46.2)	29.4(27.8, 31.0)	10.7(9.6, 11.8)	51.6(49.8, 53.3)
Peru	17.8(17.0, 18.6)	3.4(3.0, 3.8)	0.7(0.5, 0.8)	18.5(17.7, 19.3)
Rwanda	37.7(36.1, 39.3)	9.1(8.2, 10.1)	2.3(1.8, 2.8)	39.7(38.1, 41.3)
Sao Tome	29.4(27.1, 31.8)	13.2(11.5, 15.0)	10.3(8.8, 11.9)	39.3(36.8, 41.8)
Senegal	18.4(17.7, 19.1)	14.6(13.9, 15.2)	7.6(7.1, 8.1)	25.7(24.9, 26.5)
Sierra Leone	37.8(36.3, 39.3)	16.1(15.0, 17.3)	9.5(8.6, 10.4)	44.9(43.4, 46.5)
Swaziland	27.5(25.6, 29.5)	5.0(4.0, 5.9)	2.5(1.8, 3.2)	29.8(27.8, 31.7)
Tajikistan	26.1(24.8, 27.4)	12.1(11.2, 13.1)	9.9(9.1, 10.8)	35.1(33.7, 36.5)
Tanzania	34.1(33.1, 35.0)	13.6(12.9, 14.3)	4.8(4.3, 5.2)	38.5(37.5, 39.5)
Timor-Leste	57.6(56.5, 58.7)	44.2(43.1, 45.3)	18.5(17.7, 19.4)	71.1(70.0, 72.1)
Togo	26.7(25.1, 28.2)	15.7(14.5, 17.0)	6.8(5.9, 7.6)	32.3(30.7, 33.9)
Uganda	33.2(31.1, 35.2)	13.7(12.2, 15.1)	5.0(4.0, 5.9)	37.7(35.6, 39.8)
Yemen	46.3(45.5, 47.2)	38.9(38.0, 39.7)	16.4(15.7, 17.0)	58.6(57.7, 59.4)
Zambia	40.0(39.1, 40.9)	14.7(14.0, 15.3)	6.0(5.6, 6.4)	45.5(44.6, 46.4)
Zimbabwe	26.2(25.0, 27.4)	8.1(7.3, 8.8)	3.5(3.0, 4.0)	30.1(28.8, 31.4)

§ Democratic Republic of Congo

§§ Dominican Republic

Note:

1. For each country, the estimates are weighted with sample weight
2. For global estimate, we pool data from different countries together and weight with sample weight, but not population size
3. Stunting/Underweight/Wasting prevalence is calculated as the weighted number of children under-5 years old who was height-for-age/weight-for-age/height-for-weight z score less than -2 standard deviations divided by the total number of children under-5 years old

© 2022 Gausman J et al. *JAMA Network Open.*

eTable 2. Percentage of Children Under 5 Years with Single and Multiple Failure Among Those Experiencing Stunting, Underweight and Wasting Across 56 Countries

	Stunted		Underweight		Wasted	
	% of children stunted only in all stunted children	% of children stunted with other failure in all stunted children	% of children underweight only in all underweight children	% of children underweight with other failure in all underweight children	% of children wasted only in all wasted children	% of children wasted with other failure in all wasted children
Albania	85.1(80.9, 89.3)	14.9(10.7, 19.1)	8.5(2.4, 14.6)	91.5(85.4, 97.6)	74.9(66.9, 83.0)	25.1(17.0, 33.1)
Angola	57.2(55.2, 59.2)	42.8(40.8, 44.8)	7.2(5.8, 8.6)	92.8(91.4, 94.2)	29.5(24.5, 34.5)	70.5(65.5, 75.5)
Armenia	81.3(75.3, 87.2)	18.7(12.8, 24.7)	14.7(3.8, 25.5)	85.3(74.5, 96.2)	74.6(64.6, 84.6)	25.4(15.4, 35.4)
Azerbaijan	79.3(75.7, 82.8)	20.7(17.2, 24.3)	8.1(3.7, 12.5)	91.9(87.5, 96.3)	59.5(50.5, 68.5)	40.5(31.5, 49.5)
Benin	65.7(64.1, 67.3)	34.3(32.7, 35.9)	4.1(3.1, 5.0)	95.9(95.0, 96.9)	47.2(44.4, 50.0)	52.8(50.0, 55.6)
Bangladesh	34.0(32.2, 35.9)	66.0(64.1, 67.8)	9.1(7.9, 10.3)	90.9(89.7, 92.1)	22.3(19.7, 24.9)	77.7(75.1, 80.3)
Bolivia	86.7(85.2, 88.2)	13.3(11.8, 14.8)	5.8(3.3, 8.3)	94.2(91.7, 96.7)	41.5(32.3, 50.7)	58.5(49.3, 67.7)
Burkina Faso	47.9(45.9, 50.0)	52.1(50.0, 54.1)	6.1(4.9, 7.2)	93.9(92.8, 95.1)	32.2(29.3, 35.1)	67.8(64.9, 70.7)
Burundi	55.3(53.0, 57.5)	44.7(42.5, 47.0)	4.0(2.7, 5.3)	96.0(94.7, 97.3)	13.7(9.0, 18.5)	86.3(81.5, 91.0)
Cambodia	45.3(42.7, 47.9)	54.7(52.1, 57.3)	12.1(10.1, 14.2)	87.9(85.8, 89.9)	27.0(22.7, 31.2)	73.0(68.8, 77.3)
Cameroon	61.8(59.4, 64.2)	38.2(35.8, 40.6)	5.7(4.0, 7.4)	94.3(92.6, 96.0)	34.2(28.7, 39.8)	65.8(60.2, 71.3)
Chad	41.1(39.6, 42.6)	58.9(57.4, 60.4)	4.5(3.8, 5.2)	95.5(94.8, 96.2)	28.7(26.4, 31.1)	71.3(68.9, 73.6)
Comoros	63.7(60.1, 67.4)	36.3(32.6, 39.9)	7.0(4.3, 9.7)	93.0(90.3, 95.7)	46.7(40.8, 52.6)	53.3(47.4, 59.2)
Congo	63.8(61.1, 66.6)	36.2(33.4, 38.9)	10.2(7.7, 12.7)	89.8(87.3, 92.3)	53.8(47.6, 60.1)	46.2(39.9, 52.4)
Cote d'Ivoire	63.3(60.2, 66.3)	36.7(33.7, 39.8)	8.9(6.3, 11.5)	91.1(88.5, 93.7)	43.8(37.3, 50.3)	56.2(49.7, 62.7)
DRC[§]	55.6(54.0, 57.3)	44.4(42.7, 46.0)	4.2(3.3, 5.1)	95.8(94.9, 96.7)	37.4(33.6, 41.1)	62.6(58.9, 66.4)
DR[§§]	69.7(63.9, 75.6)	30.3(24.4, 36.1)	16.3(9.5, 23.0)	83.7(77.0, 90.5)	52.4(40.8, 64.0)	47.6(36.0, 59.2)
Egypt	84.2(82.8, 85.6)	15.8(14.4, 17.2)	5.8(4.3, 7.4)	94.2(92.6, 95.7)	71.6(69.3, 74.0)	28.4(26.0, 30.7)
Ethiopia	50.2(48.5, 51.9)	49.8(48.1, 51.5)	4.8(3.9, 5.6)	95.2(94.4, 96.1)	36.1(33.2, 39.0)	63.9(61.0, 66.8)
Gambia	55.5(52.1, 59.0)	44.5(41.0, 47.9)	8.2(5.9, 10.5)	91.8(89.5, 94.1)	43.8(38.7, 48.9)	56.2(51.1, 61.3)
Gabon	72.3(69.1, 75.4)	27.7(24.6, 30.9)	6.4(3.5, 9.3)	93.6(90.7, 96.5)	49.7(41.2, 58.2)	50.3(41.8, 58.8)

© 2022 Gausman J et al. *JAMA Network Open.*

Ghana	59.7(55.5, 63.9)	40.3(36.1, 44.5)	18.4(13.9, 22.8)	81.6(77.2, 86.1)	37.2(28.9, 45.5)	62.8(54.5, 71.1)
Guatemala	74.4(73.2, 75.5)	25.6(24.5, 26.8)	3.1(2.3, 4.0)	96.9(96.0, 97.7)	9.3(3.0, 15.7)	90.7(84.3, 97.0)
Guinea	56.7(53.6, 59.9)	43.3(40.1, 46.4)	4.3(2.6, 6.1)	95.7(93.9, 97.4)	37.3(31.9, 42.8)	62.7(57.2, 68.1)
Guyana	60.2(55.1, 65.4)	39.8(34.6, 44.9)	11.5(6.3, 16.7)	88.5(83.3, 93.7)	42.5(31.2, 53.8)	57.5(46.2, 68.8)
Haiti	60.7(57.5, 63.9)	39.3(36.1, 42.5)	7.8(5.4, 10.2)	92.2(89.8, 94.6)	27.2(21.0, 33.4)	72.8(66.6, 79.0)
Honduras	73.7(72.0, 75.4)	26.3(24.6, 28.0)	7.9(6.0, 9.8)	92.1(90.2, 94.0)	32.8(25.0, 40.5)	67.2(59.5, 75.0)
India	35.0(34.6, 35.3)	65.0(64.7, 65.4)	7.1(6.9, 7.3)	92.9(92.7, 93.1)	29.4(29.0, 29.8)	70.6(70.2, 71.0)
Jordan	77.6(74.1, 81.1)	22.4(18.9, 25.9)	16.4(11.1, 21.7)	83.6(78.3, 88.9)	56.3(48.0, 64.6)	43.7(35.4, 52.0)
Kenya	67.3(66.0, 68.6)	32.7(31.4, 34.0)	7.3(6.3, 8.4)	92.7(91.6, 93.7)	33.7(30.8, 36.6)	66.3(63.4, 69.2)
Kyrgyzstan	87.2(84.7, 89.6)	12.8(10.4, 15.3)	4.3(0.9, 7.6)	95.7(92.4, 99.1)	56.3(47.1, 65.4)	43.7(34.6, 52.9)
Lesotho	72.4(68.3, 76.6)	27.6(23.4, 31.7)	5.5(1.8, 9.2)	94.5(90.8, 98.2)	29.2(15.5, 42.8)	70.8(57.2, 84.5)
Liberia	59.1(56.1, 62.2)	40.9(37.8, 43.9)	5.3(3.3, 7.3)	94.7(92.7, 96.7)	42.0(35.2, 48.7)	58.0(51.3, 64.8)
Malawi	72.8(70.8, 74.9)	27.2(25.1, 29.2)	3.5(2.0, 4.9)	96.5(95.1, 98.0)	34.4(27.0, 41.7)	65.6(58.3, 73.0)
Maldives	45.7(41.0, 50.3)	54.3(49.7, 59.0)	15.1(11.7, 18.5)	84.9(81.5, 88.3)	37.3(31.6, 43.1)	62.7(56.9, 68.4)
Mali	48.1(45.7, 50.5)	51.9(49.5, 54.3)	3.5(2.4, 4.6)	96.5(95.4, 97.6)	30.9(27.0, 34.8)	69.1(65.2, 73.0)
Mozambique	70.8(69.3, 72.3)	29.2(27.7, 30.7)	4.3(3.2, 5.4)	95.7(94.6, 96.8)	41.1(36.7, 45.5)	58.9(54.5, 63.3)
Myanmar	51.8(49.0, 54.5)	48.2(45.5, 51.0)	11.6(9.4, 13.9)	88.4(86.1, 90.6)	35.7(30.1, 41.4)	64.3(58.6, 69.9)
Namibia	57.5(52.3, 62.6)	42.5(37.4, 47.7)	13.6(9.0, 18.2)	86.4(81.8, 91.0)	44.0(35.1, 52.8)	56.0(47.2, 64.9)
Nepal	43.7(40.5, 46.8)	56.3(53.2, 59.5)	6.9(5.0, 8.8)	93.1(91.2, 95.0)	28.0(22.4, 33.6)	72.0(66.4, 77.6)
Niger	36.2(34.1, 38.3)	63.8(61.7, 65.9)	6.0(4.8, 7.1)	94.0(92.9, 95.2)	23.0(20.2, 25.8)	77.0(74.2, 79.8)
Pakistan	44.5(41.8, 47.1)	55.5(52.9, 58.2)	5.2(3.7, 6.8)	94.8(93.2, 96.3)	22.7(18.1, 27.4)	77.3(72.6, 81.9)
Peru	83.6(81.9, 85.3)	16.4(14.7, 18.1)	6.4(4.0, 8.9)	93.6(91.1, 96.0)	24.8(13.7, 35.9)	75.2(64.1, 86.3)
Rwanda	79.4(77.2, 81.6)	20.6(18.4, 22.8)	5.2(2.8, 7.7)	94.8(92.3, 97.2)	29.5(19.2, 39.9)	70.5(60.1, 80.8)
Sao Tome Principe	71.5(67.1, 75.9)	28.5(24.1, 32.9)	9.5(5.3, 13.6)	90.5(86.4, 94.7)	49.1(41.7, 56.5)	50.9(43.5, 58.3)
Senegal	47.9(45.9, 49.9)	52.1(50.1, 54.1)	11.9(10.4, 13.3)	88.1(86.7, 89.6)	29.9(27.0, 32.9)	70.1(67.1, 73.0)

© 2022 Gausman J et al. *JAMA Network Open.*

Sierra Leone	66.9(64.5, 69.3)	33.1(30.7, 35.5)	4.8(3.2, 6.5)	95.2(93.5, 96.8)	37.0(32.1, 41.9)	63.0(58.1, 67.9)
Swaziland	84.7(81.7, 87.8)	15.3(12.2, 18.3)	2.9(-0.5, 6.3)	97.1(93.7, 100.5)	56.2(42.1, 70.3)	43.8(29.7, 57.9)
Tajikistan	70.8(68.1, 73.5)	29.2(26.5, 31.9)	6.3(4.3, 8.4)	93.7(91.6, 95.7)	45.1(40.5, 49.7)	54.9(50.3, 59.5)
Tanzania	66.9(65.2, 68.6)	33.1(31.4, 34.8)	6.3(4.9, 7.6)	93.7(92.4, 95.1)	37.7(33.1, 42.3)	62.3(57.7, 66.9)
Timor-Leste	38.1(36.6, 39.5)	61.9(60.5, 63.4)	4.6(3.9, 5.3)	95.4(94.7, 96.1)	26.7(24.4, 28.9)	73.3(71.1, 75.6)
Togo	53.1(49.8, 56.4)	46.9(43.6, 50.2)	8.1(5.8, 10.5)	91.9(89.5, 94.2)	35.4(29.2, 41.6)	64.6(58.4, 70.8)
Uganda	67.0(63.4, 70.6)	33.0(29.4, 36.6)	7.0(4.1, 10.0)	93.0(90.0, 95.9)	37.2(28.1, 46.3)	62.8(53.7, 71.9)
Yemen	35.0(33.8, 36.2)	65.0(63.8, 66.2)	6.5(5.8, 7.2)	93.5(92.8, 94.2)	21.3(19.6, 23.0)	78.7(77.0, 80.4)
Zambia	69.9(68.6, 71.2)	30.1(28.8, 31.4)	6.4(5.2, 7.5)	93.6(92.5, 94.8)	48.6(45.0, 52.3)	51.4(47.7, 55.0)
Zimbabwe	76.2(73.9, 78.6)	23.8(21.4, 26.1)	9.5(6.5, 12.4)	90.5(87.6, 93.5)	42.3(34.8, 49.8)	57.7(50.2, 65.2)

[§] Democratic Republic of Congo
[§§] Dominican Republic

© 2022 Gausman J et al. *JAMA Network Open.*

eTable 3. Percentage of Children Under 5 Experiencing Individual and Concurrent Anthropometric Failures According to the Categories of Anthropometric Failure Across 56 Countries

Country	Stunted only (%)	Underweight only (%)	Wasted only (%)	Stunted and underweight, but not wasted (%)	Underweight and wasted, but not stunted (%)	Stunted, underweight, and wasted (%)
**Global**	**16.8(16.7, 16.9)**	**1.7(1.7, 1.7)**	**4.2(4.1, 4.2)**	**13.9(13.8, 14.0)**	**5.0(4.9, 5.0)**	**4.1(4.1, 4.2)**
Albania	16.4(14.4, 18.4)	0.4(0.1, 0.8)	6.8(5.4, 8.1)	2.5(1.6, 3.3)	1.8(1.1, 2.6)	0.4(0.1, 0.8)
Angola	21.4(20.4, 22.4)	1.4(1.1, 1.7)	1.5(1.2, 1.8)	13.4(12.6, 14.2)	1.5(1.2, 1.8)	2.0(1.7, 2.4)
Armenia	7.8(6.5, 9.1)	0.4(0.1, 0.7)	3.2(2.3, 4.1)	1.0(0.5, 1.4)	0.6(0.2, 1.0)	0.2(-0.0, 0.5)
Azerbaijan	19.9(18.1, 21.7)	0.6(0.3, 1.0)	4.1(3.2, 5.0)	4.3(3.4, 5.2)	1.9(1.3, 2.5)	0.9(0.5, 1.3)
Benin	28.7(27.7, 29.7)	0.8(0.6, 1.0)	7.6(7.0, 8.2)	11.4(10.7, 12.1)	4.9(4.4, 5.4)	3.6(3.2, 4.0)
Bangladesh	12.3(11.6, 13.1)	3.0(2.6, 3.4)	3.2(2.8, 3.6)	18.5(17.6, 19.4)	5.8(5.2, 6.3)	5.4(4.9, 5.9)
Bolivia	23.5(22.5, 24.4)	0.2(0.1, 0.4)	0.6(0.4, 0.8)	3.2(2.8, 3.6)	0.4(0.3, 0.6)	0.4(0.3, 0.6)
Burkina Faso	16.5(15.6, 17.4)	1.6(1.3, 1.9)	5.1(4.6, 5.6)	13.4(12.6, 14.2)	6.1(5.6, 6.7)	4.6(4.1, 5.1)
Burundi	32.0(30.4, 33.5)	1.1(0.8, 1.5)	0.8(0.5, 1.1)	22.4(21.0, 23.8)	1.5(1.1, 1.9)	3.5(2.8, 4.1)
Cambodia	14.5(13.4, 15.5)	2.9(2.4, 3.4)	2.6(2.1, 3.1)	14.1(13.1, 15.1)	3.7(3.2, 4.3)	3.4(2.8, 3.9)
Cameroon	19.7(18.6, 20.8)	0.8(0.6, 1.1)	2.0(1.6, 2.4)	9.9(9.0, 10.7)	1.5(1.2, 1.9)	2.4(1.9, 2.8)
Chad	16.3(15.5, 17.0)	1.3(1.1, 1.5)	3.8(3.4, 4.2)	18.1(17.3, 18.8)	4.2(3.8, 4.6)	5.3(4.8, 5.7)
Comoros	18.9(17.3, 20.4)	1.1(0.7, 1.5)	5.2(4.3, 6.1)	8.6(7.5, 9.7)	3.8(3.0, 4.5)	2.2(1.6, 2.7)
Congo	14.8(13.7, 15.8)	1.1(0.8, 1.4)	3.0(2.5, 3.5)	7.4(6.6, 8.2)	1.6(1.3, 2.0)	0.9(0.7, 1.2)
Cote d'Ivoire	18.8(17.5, 20.2)	1.3(0.9, 1.7)	3.4(2.8, 4.0)	9.0(8.0, 10.0)	2.5(1.9, 3.0)	1.9(1.4, 2.4)
DRC§	23.5(22.6, 24.5)	0.9(0.7, 1.2)	3.0(2.6, 3.3)	16.4(15.6, 17.3)	2.6(2.3, 3.0)	2.3(2.0, 2.7)
DR§§	4.8(4.0, 5.5)	0.5(0.3, 0.8)	1.0(0.6, 1.3)	1.8(1.4, 2.3)	0.7(0.4, 0.9)	0.2(0.1, 0.4)
Egypt	18.1(17.4, 18.7)	0.3(0.2, 0.4)	6.1(5.7, 6.5)	2.8(2.5, 3.1)	1.8(1.6, 2.0)	0.6(0.5, 0.7)
Ethiopia	19.5(18.6, 20.3)	1.2(0.9, 1.4)	3.7(3.3, 4.1)	15.8(15.0, 16.5)	3.3(3.0, 3.7)	3.1(2.7, 3.4)
Gambia	13.4(12.2, 14.6)	1.3(0.9, 1.7)	5.4(4.6, 6.2)	8.0(7.1, 9.0)	4.2(3.5, 4.9)	2.7(2.2, 3.3)
Gabon	11.6(10.5, 12.7)	0.4(0.2, 0.6)	1.7(1.3, 2.2)	3.7(3.1, 4.4)	1.0(0.7, 1.4)	0.7(0.4, 1.0)
Ghana	10.7(9.5, 11.9)	2.0(1.5, 2.5)	1.8(1.3, 2.3)	5.9(5.0, 6.8)	1.6(1.1, 2.1)	1.4(0.9, 1.8)
Guatemala	34.6(33.8, 35.5)	0.4(0.3, 0.5)	0.1(0.0, 0.1)	11.5(10.9, 12.1)	0.2(0.1, 0.3)	0.5(0.3, 0.6)

© 2022 Gausman J et al. *JAMA Network Open.*

Guinea	17.5(16.2, 18.9)	0.8(0.5, 1.1)	3.7(3.0, 4.4)	11.0(9.9, 12.1)	3.8(3.2, 4.5)	2.4(1.9, 2.9)
Guyana	11.2(9.7, 12.8)	1.2(0.7, 1.8)	2.3(1.5, 3.0)	6.4(5.2, 7.6)	2.1(1.3, 2.8)	1.0(0.5, 1.5)
Haiti	12.7(11.6, 13.7)	0.9(0.6, 1.2)	1.4(1.0, 1.8)	6.7(5.9, 7.4)	2.2(1.8, 2.7)	1.5(1.1, 1.9)
Honduras	16.5(15.7, 17.2)	0.5(0.4, 0.7)	0.4(0.3, 0.6)	5.5(5.0, 5.9)	0.5(0.4, 0.6)	0.4(0.3, 0.5)
India	13.4(13.3, 13.6)	2.5(2.5, 2.6)	6.2(6.1, 6.3)	18.3(18.2, 18.5)	8.2(8.1, 8.3)	6.6(6.5, 6.7)
Jordan	5.9(5.4, 6.5)	0.5(0.3, 0.7)	1.4(1.1, 1.7)	1.5(1.2, 1.8)	0.8(0.6, 1.0)	0.3(0.1, 0.4)
Kenya	17.4(16.8, 17.9)	0.8(0.7, 0.9)	1.4(1.2, 1.5)	7.2(6.8, 7.5)	1.4(1.2, 1.6)	1.3(1.1, 1.4)
Kyrgyzstan	15.5(14.4, 16.6)	0.1(0.0, 0.3)	1.5(1.1, 1.9)	1.9(1.5, 2.4)	0.8(0.6, 1.1)	0.3(0.2, 0.5)
Lesotho	23.5(21.2, 25.8)	0.6(0.2, 1.0)	0.9(0.4, 1.4)	8.1(6.6, 9.5)	1.3(0.7, 1.9)	0.9(0.4, 1.4)
Liberia	17.9(16.5, 19.2)	0.8(0.5, 1.1)	2.7(2.1, 3.2)	10.5(9.4, 11.6)	1.8(1.4, 2.3)	1.9(1.4, 2.3)
Malawi	26.8(25.6, 28.0)	0.4(0.2, 0.6)	1.0(0.7, 1.2)	8.8(8.0, 9.6)	0.8(0.6, 1.1)	0.9(0.7, 1.2)
Maldives	8.2(7.1, 9.3)	2.5(1.9, 3.2)	4.0(3.2, 4.8)	7.5(6.5, 8.6)	4.5(3.7, 5.3)	2.2(1.6, 2.8)
Mali	18.5(17.3, 19.6)	0.9(0.6, 1.2)	4.0(3.4, 4.5)	15.7(14.6, 16.8)	4.6(4.0, 5.2)	4.3(3.7, 4.9)
Mozambique	30.3(29.4, 31.2)	0.6(0.5, 0.8)	2.4(2.1, 2.8)	10.8(10.2, 11.4)	1.8(1.5, 2.1)	1.7(1.5, 2.0)
Myanmar	15.1(14.0, 16.2)	2.3(1.8, 2.7)	2.6(2.1, 3.1)	12.2(11.2, 13.2)	3.0(2.5, 3.6)	1.6(1.2, 2.0)
Namibia	12.7(11.0, 14.3)	1.9(1.2, 2.5)	3.4(2.5, 4.3)	7.5(6.2, 8.8)	2.4(1.7, 3.2)	1.9(1.2, 2.5)
Nepal	17.6(16.0, 19.1)	2.0(1.4, 2.5)	3.1(2.4, 3.8)	18.7(17.1, 20.3)	3.9(3.1, 4.7)	4.0(3.2, 4.8)
Niger	15.7(14.6, 16.7)	2.2(1.7, 2.6)	4.2(3.6, 4.7)	20.1(18.9, 21.2)	6.4(5.7, 7.1)	7.5(6.8, 8.3)
Pakistan	19.7(18.3, 21.2)	1.5(1.1, 2.0)	2.4(1.9, 3.0)	19.6(18.2, 21.0)	3.2(2.6, 3.8)	5.1(4.3, 5.8)
Peru	14.9(14.2, 15.6)	0.2(0.1, 0.3)	0.2(0.1, 0.2)	2.7(2.4, 3.0)	0.3(0.2, 0.4)	0.2(0.1, 0.3)
Rwanda	29.9(28.4, 31.5)	0.5(0.3, 0.7)	0.7(0.4, 0.9)	7.0(6.2, 7.9)	0.9(0.6, 1.2)	0.7(0.4, 1.0)
Sao Tome	21.0(18.9, 23.1)	1.3(0.7, 1.8)	5.1(3.9, 6.2)	6.7(5.4, 8.0)	3.6(2.6, 4.6)	1.6(1.0, 2.3)
Senegal	8.8(8.3, 9.3)	1.7(1.5, 2.0)	2.3(2.0, 2.5)	7.5(7.1, 8.0)	3.2(2.9, 3.6)	2.1(1.8, 2.3)
Sierra Leone	25.3(23.9, 26.6)	0.8(0.5, 1.1)	3.5(2.9, 4.1)	9.4(8.5, 10.3)	2.8(2.3, 3.4)	3.1(2.6, 3.6)
Swaziland	23.3(21.5, 25.2)	0.1(-0.0, 0.3)	1.4(0.9, 1.9)	3.7(2.9, 4.6)	0.6(0.3, 1.0)	0.5(0.2, 0.7)
Tajikistan	18.5(17.3, 19.6)	0.8(0.5, 1.0)	4.5(3.9, 5.1)	5.9(5.2, 6.6)	3.8(3.2, 4.3)	1.7(1.3, 2.1)
Tanzania	22.9(22.1, 23.8)	0.9(0.7, 1.1)	1.8(1.5, 2.1)	9.6(9.0, 10.2)	1.4(1.2, 1.7)	1.5(1.3, 1.8)

© 2022 Gausman J et al. *JAMA Network Open.*

Timor-Leste	21.9(21.0, 22.9)	2.0(1.7, 2.4)	4.9(4.5, 5.4)	28.6(27.5, 29.6)	6.5(5.9, 7.0)	7.1(6.5, 7.7)
Togo	14.2(13.0, 15.4)	1.3(0.9, 1.7)	2.4(1.9, 2.9)	10.1(9.0, 11.1)	1.9(1.5, 2.4)	2.4(1.9, 3.0)
Uganda	22.2(20.4, 24.0)	1.0(0.5, 1.4)	1.9(1.3, 2.4)	9.6(8.3, 10.8)	1.8(1.2, 2.3)	1.4(0.9, 1.9)
Yemen	16.2(15.6, 16.8)	2.5(2.3, 2.8)	3.5(3.2, 3.8)	23.5(22.7, 24.2)	6.2(5.8, 6.6)	6.7(6.2, 7.1)
Zambia	27.9(27.1, 28.8)	0.9(0.8, 1.1)	2.9(2.6, 3.2)	10.7(10.1, 11.2)	1.7(1.5, 1.9)	1.4(1.2, 1.6)
Zimbabwe	20.2(19.0, 21.3)	0.8(0.5, 1.0)	1.5(1.2, 1.8)	5.0(4.4, 5.6)	1.3(1.0, 1.6)	0.7(0.5, 1.0)

§ Democratic Republic of Congo

§§ Dominican Republic

Note:

1. For each country, the estimates are weighted with sample weight
2. For global estimate, we pool data from different countries together and weight with sample weight, but not population size
3. “No failure” is calculated as the weighted number of children under-5 years old who were not stunted, underweight, or wasted divided by the total number of children under-5 years old
4. “Stunted only” is calculated as the weighted number of children under-5 years old who were stunted but not underweight or wasted divided by the total number of children under-5 years old
5. “Underweight only” is calculated as the weighted number of children under-5 years old who were underweight but not stunted or wasted divided by the total number of children under-5 years old
6. “Wasting only” is calculated as the weighted number of children under-5 years old who were wasted but not stunted or underweight divided by the total number of children under-5 years old
7. “Stunted and underweight, but not wasted” is calculated as the weighted number of children under-5 years old who were stunted and underweight but not wasted divided by the total number of children under-5 years old
8. “Underweight and wasted, but not stunted” is calculated as the weighted number of children under-5 years old who were underweight and wasted but not stunted divided by the total number of children under-5 years old
9. “Stunted, underweight, and wasted” is calculated as the weighted number of children under-5 years old who were stunted, underweight, and wasted divided by the total number of children under-5 years old

© 2022 Gausman J et al. *JAMA Network Open.*

eTable 4. Summary of Current Approaches for Estimating Undernutrition and Their Advantages and Disadvantages

Approach	Purpose / Underlying Constructs	Definition	Advantages	Disadvantages
**Approach 1**<br>World Health Organization (WHO) Indicators for Malnutrition in Children	*Stunting*<br>Indicator of chronic malnutrition; understood to relate to duration of malnutrition<br><br>*Underweight*<br>Indicator of stunting or wasting or combination and typically used to reflect the extent of both chronic and acute malnutrition<br><br>*Wasting*<br>Indicator of acute malnutrition	*Stunting*<br>Low length/height for age<br>(-2 SD of the WHO Child Growth Standards median)<br><br>*Underweight*<br>Low weight for age<br>(-2 SD of the WHO Child Growth Standards median)<br><br>*Wasting*<br>Low weight for height<br>(-2 SD of the WHO Child Growth Standards median)	*General*<br>- Unnecessary to assess caloric intake<br>- Can provide a comprehensive overview of concentration of malnutrition based on age, gender, location of inhabitance, etc.<br>- Universal for children below the age of 5<br><br>*Stunting*<br>- Offers picture of past nutritional history and not just current nutritional status<br>- Useful for serial measurements<br><br>*Underweight*<br>- Simple<br>- Useful for serial measurements<br><br>*Wasting*<br>- Can be used when age of child is unknown or unreliable<br>- Useful for serial measurements<br>- Can also be used to determine classification of children as overweight or obese	*General*<br>- Cannot provide a single figure of the overall estimate of malnutrition among children in a population<br>- Failure according to the norms is not sufficient for diagnosis as malnourished – underlying cause is not revealed<br><br>*Stunting*<br>- Does not consider children who are classified as either underweight only or wasting only<br>- In many communities, ages of children are not known<br><br>*Underweight*<br>- Does not consider children who are classified as either stunting only or wasting only<br>- In many communities, ages of children are not known<br>- Does not distinguish between acute and chronic forms of undernutrition<br>- A composite indicator and thus can be difficult to interpret<br><br>*Wasting*

© 2022 Gausman J et al. *JAMA Network Open.*

				- Does not consider children who are classified as either stunted only or underweight only
**Approach 2**<br>Svedberg Composite Index of Anthropometric Failure (CIAF) Measure of Child Malnutrition	A single inclusive measure of child malnutrition that accounts for all children experiencing any combination (i.e., single failure, double failure, or triple failure) of anthropometric failures (i.e., stunting, underweight, or wasting)	CIAF<br>= (B) + (C) + (D) + (E) + (F) + (G)<br>= 1 – (A)<br><br>(A) No failure<br>(B) Wasting only<br>(C) Wasting and underweight<br>(D) Wasting, stunting, and underweight<br>(E) Stunting and underweight<br>(F) Stunting only<br>(G) Underweight only	- Allows for single measure of the burden of undernutrition<br><br>- Serves as a more comprehensive indicator of malnutrition than the conventional WHO indices<br><br>- Conveys information about the dominating failures, which in turn impacts policy and allows for targeted interventions<br><br>- Sources of anthropometric data are readily available to	- Does not address need for a composite anthropometric and clinical classification that can be used for both clinical work and community health work<br><br>- Also captures children who are primarily ill for reasons unrelated to nutrition

© 2022 Gausman J et al. *JAMA Network Open.*

			researchers on the Internet	
**Approach 3** Categories of Anthropometric Failure (CAF)	Separation of CIAF data into seven categories of child malnutrition	*Classification categories*<br>(A) No failure<br>(B) Wasting only<br>(C) Wasting and underweight<br>(D) Wasting, stunting, and underweight<br>(E) Stunting and underweight<br>(F) Stunting only<br>(G) Underweight only	- Disaggregated data can be subject to further analyses<br><br>- Enables the identification of undernourished children that are missed by the conventional WHO indices<br><br>- Allows for examination of the relationship between malnutrition, morbidity, mortality, and poverty	-Does not provide a single, easily interpretable estimate of undernutrition

© 2022 Gausman J et al. *JAMA Network Open.*

eFigure 1. Comparison of Different Approaches to Measuring Undernutrition by Anthropometric Failure (AF): Conventional Categorization of AF, the Composite Index of AF (CIAF) and the Categories of AF (CAF)

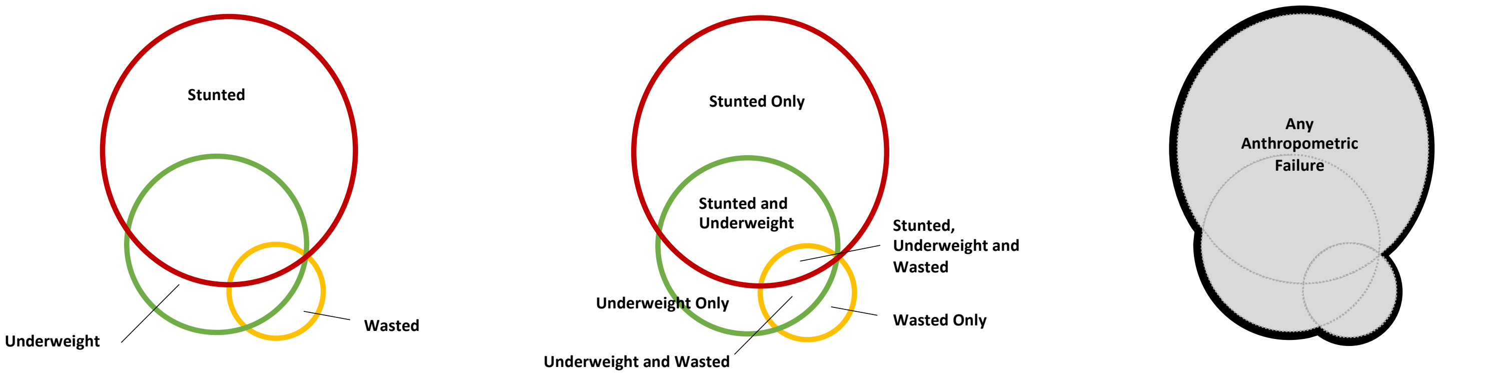

Conventional Anthropometric Measures

Categories of Anthropometric Failure (CAF)

Composite Index of Anthropometric Failure (CIAF)

© 2022 Gausman J et al. *JAMA Network Open.*
